# Effectiveness of tori line use to reduce seabird bycatch in pelagic longline fishing

**DOI:** 10.1371/journal.pone.0184465

**Published:** 2017-09-08

**Authors:** Andrés Domingo, Sebastián Jiménez, Martin Abreu, Rodrigo Forselledo, Oliver Yates

**Affiliations:** 1 Laboratorio de Recursos Pelágicos, Dirección Nacional de Recursos Acuáticos, Montevideo, Uruguay; 2 Albatross Task Force, Proyecto Albatros y Petreles – Uruguay, Centro de Investigación y Conservación Marina (CICMAR), Canelones, Uruguay; 3 BirdLife International Marine Programme, RSPB, The Lodge, Sandy, Bedfordshire, United Kingdom; Hawaii Pacific University, UNITED STATES

## Abstract

Industrial longline fisheries cause the death of large numbers of seabirds annually. Various mitigation measures have been proposed, including the use of tori lines. In this study the efficiency of a single tori line to reduce seabird bycatch was tested on pelagic longline vessels (25-37m length). Thirteen fishing trips were carried out in the area and season of the highest bycatch rates recorded in the southwest Atlantic (2009–2011). We deployed two treatments in random order: sets with a tori line and without a tori line (control treatment). The use of a tori line significantly reduced seabird bycatch rates. Forty three and seven birds were captured in the control (0.85 birds/1,000 hooks, n = 49 sets) and in the tori line treatment (0.13 birds/1,000 hooks, n = 51 sets), respectively. In 47% of the latter sets the tori line broke either because of entanglement with the longline gear or by tension. This diminished the tori line effectiveness; five of the seven captures during sets where a tori line was deployed were following ruptures. Nine additional trips were conducted with a tori line that was modified to reduce entanglements (2012–2016). Seven entanglements were recorded in 73 longline sets. The chance of a rupture on these trips was 4% (95% c.l. = 1–18%) of that during 2009–2011. This work shows that the use of a tori line reduces seabird bycatch in pelagic longline fisheries and is a practice suitable for medium size vessels (~25-40m length). Because the study area has historically very high bycatch rates at global level, this tori line design is potentially useful to reduce seabird bycatch in many medium size pelagic longline vessel fishing in the southern hemisphere.

## Introduction

The incidental mortality (bycatch) in industrial fisheries is the most pervasive threat for albatrosses and large petrels [1]. Bycatch estimates for all seabird species in longline fisheries indicate that over a hundred thousand birds are caught each year, a large proportion of which are albatrosses and petrels [2]. Various mitigation measures have been proposed [3–6], including the use of a tori line [7, 8]; i.e., a line towed from a high point at the aft of a vessel, from which several streamers are attached, to scare seabirds and prevent their access to the critical area where baited hooks sink. The effectiveness of tori lines to reduce seabird bycatch has been successfully tested in several demersal fisheries (reviewed in [3]). Similar research in pelagic longline fisheries has been partially limited by the operational challenges of deploying such devices over a slow sinking fishing line. This is especially problematic in small (< 25m long) to medium size vessels (~ 25–40m) where entanglement of the tori line with the fishing line (including branch lines and buoy lines) is frequent. Though experimental research in these fisheries has included some vessels of over 30 m in the North Pacific [9, 10], most studies in both hemispheres are based on large (> 40m) fishing [6, 9–13] and research [14] vessels. These studies have compared the performance of different tori line designs, using either single [9, 13] or paired tori lines [11], or both [10]. Most recent experiments included the combined use of weighted and unweighted branch lines with either a single tori line [12] or paired tori lines in diurnal and nocturnal setting [6]. Only an earlier study in the central North Pacific fishing grounds experimentally compared the effectiveness of a single tori line *vs* a control treatment [14], using seabird attacks on baited hooks as a proxy of bycatch. Therefore, there is a lack of experimental studies on tori line effectiveness on small to medium size pelagic longline vessels, and especially in areas of high seabird-longline interactions of the southern hemisphere, where mitigation measures are urgently needed.

Pelagic longline fisheries in the southwest Atlantic are a major threat for the conservation of various species of globally threatened albatrosses and petrels [15–17]. The shelf break off Uruguay has the highest historical seabird bycatch figures recorded for the region [15, 18]. The use of tori lines as a seabird bycatch mitigation measure became mandatory in 1997 for all longline vessels flagged in Uruguay and operating inside and outside of its Economic Exclusive Zone (EEZ), pursuant to Resolution 248/997, of the “Ministerio de Ganadería Agricultura y Pesca” (MGAP). However, a study in 2004–2007 indicated that tori lines were used in less than 1% of the fishing sets of the pelagic longline fishery [16], a situation which continued until the beginning of this study [19]. Reasons for low compliance included the reluctance of captains to use a measure that causes entanglements, and hinders fishing practices. Moreover, at the time there were no tori lines scientifically tested for pelagic longline vessels, particularly for small and medium size vessels. Although tori lines were developed in large Japanese pelagic longline vessels [7], specific technical requirements were first described for demersal fisheries within the CCAMLR Convention Area [20]. To increase the level of compliance with the aforementioned resolution it was necessary to develop and test a specific tori line design that could be used with minimal problems. In this study the efficiency of a tori line to reduce incidental seabird bycatch was tested in the Uruguayan pelagic longline fleet. To accomplish this, an experiment was conducted in two phases: the first in 13 trips over the period 2009–2011 and the second in a further nine trips over the period 2012–2016. In the first phase the objective was to experimentally test a specific tori line design to reduce seabird bycatch in small to medium size longliners. The second phase was aimed at minimizing the entanglement and rupture rate of tori lines detected during the first phase.

## Materials and methods

**Fig 1:**
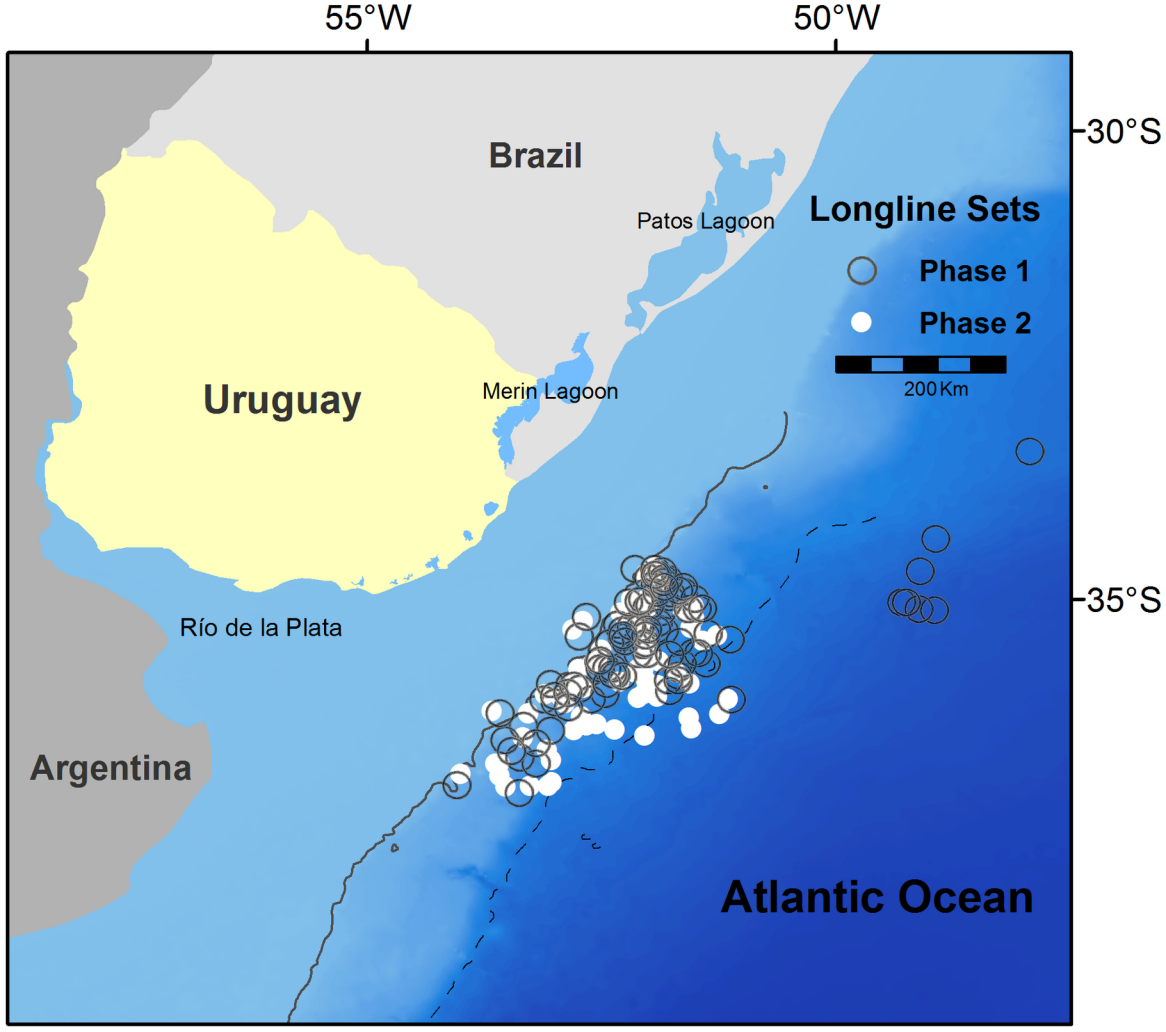
Distribution of the longline fishing sets conducted during the first (2009–2011) and second (2012–2016) phase. The 200 m (solid line) and the 3000 m (dashed line) isobaths are represented.

The experiment to test the effectiveness of the tori line (hereafter: phase 1) was conducted over the shelf break and slope off Uruguay (Fig 1) during the period of the year (i.e. May-November) where, historically, the majority of seabird bycatch events have been recorded in the southwest Atlantic [15]. Thirteen trips were made between August 2009 and November 2011. Ten of these trips (August and November 2009; August, October and November 2010; May, July, October and November 2011) were on three different commercial fishing vessels (F/V) of 25–28 m length and the remaining three (August 2009, October 2010 and July 2011) were on a research vessel (R/V) of 37 m length. The second part of the research (hereafter: phase 2), which aimed to reduce the entanglement and rupture rate of the tori line, was carried out in the same area from 2012 to 2016 (Fig 1). Nine trips were conducted; three on one of the F/V mentioned above (March, June-July and September-October of 2012) and the other six on the R/V (July 2012; June and August 2013; September 2014; December 2015, June-July 2016).

This research was conducted according to applicable national and international guidelines [19] and approved by DINARA (“Plan de Campaña de Grandes Pelágicos”: 2009, 2010, 2011, 2012, 2013–01, 2013–03, 2014, 2015, 2016–01). In phase 1 of the experiment, sets conducted with a tori line deployed were tested against sets without a tori line. This was not considered lethal experimentation (i.e. when an experiment may elevate seabird deaths above the level of bycatch that would have occurred under normal fishing operations [19]) because, as stated in the introduction, non-compliance with tori lines was standard practice in the fishery at the time. The commission on ethics in the use of animals (“Comisión de Ética del Uso de Animales”, CEUA) of “Dirección Nacional de Recursos Acuáticos” (DINARA) was not created until the end of this phase, and thus an approval was not required. Phase 2 did not require an approval of an animal ethical committee as we were recording tori line entanglement rates and all longline sets during this phase included the use of a tori line. A seabird bycatch threshold of 50 birds was established according national guidelines [19]).

### Fishing gear and operation

The fishing gear was American-style longline, with a polyamide monofilament mainline [15, 21]. The configuration of the fishing gear and operation on all vessels was consistent with the standard commercial fishery practices. Fishing gear was composed of units of five branch lines suspended on the main line between two buoys. Branch lines were made of two lengths of 2.0 mm diameter polyamide monofilament, one extending from the main line to a 75g swivel and the other extending on average 4.5 m (2.5 fathoms, range ~ 3.6–5.4 m or 2–3 fathoms) from the swivel to the hook (J type 9/0). The mean total length of the branch lines was 13.3 m (range = 12.6–14.4 m). On the R/V we constructed a similar branch line of 12.9 m long with a distance of 4.5 m between the swivel and the hook. Four types of buoys were used (foam bullets, rigid floats, polyform inflatable buoys, and radio buoys). Each section of the fishing gear (150–200 hooks) was delimited by a radio buoy that transmits a radio signal to facilitate recovery of the gear before hauling. The radio buoys consisted of a cylindrical stainless steel body, surrounded by a circular foam float, with a 4 m fiberglass antenna. The hooks were set from a central position on the stern of the vessel and deployed aft towards the port side or into the wash, mostly after sunset (night setting; see below). During phase 1, the main bait utilised was squid that had been thawed a few hours before setting. Mackerel or a mix of mackerel and squid were also used on a few sets on a F/V. During phase 2, sardine and squid or squid only were used as bait on the F/V and R/V trips, respectively.

In order to try to replicate the operations of the commercial longline fleet in all trips made by the R/V, we engaged the services of a fishing master, a boatswain and deck crew members, all of them experienced in the commercial fleet. The average speed of the vessels was 8.1 knots (range 6.9 to 9.8 knots) and varied between 7.0 and 9.8 knots in the F/V (mean = 8.0 knots) and between 6.9 and 9.3 knots in the R/V (mean = 8.1 knots). In both cases, the interval between hook deployments was typically 12 s (range 10 to 14 s). The main difference between the R/V and the F/V was the number of hooks in each set (R/V: 360–450 hooks; F/V: 700–1550 hooks). Particularly during phase 2 and only on the R/V, other weight regimes for branch lines (either 60 or 65 g within 1m of the hook) were used in some sections of the longline as part of a separate study. Although this may have affected seabird bycatch rates, data from phase 2 were only used to study tori line entanglements rates. The total length of these branch lines was kept at 12.9m; therefore, changes in the entanglement rates between phases were unlikely to be associated with this modification. A few 18/0 circle hooks were used on the R/V during phase 1. However, these hooks have no demonstrated effect on seabird bycatch in this fishery [21].

### Phase 1. Effectiveness experiment

#### Tori line design

**Fig 2:**
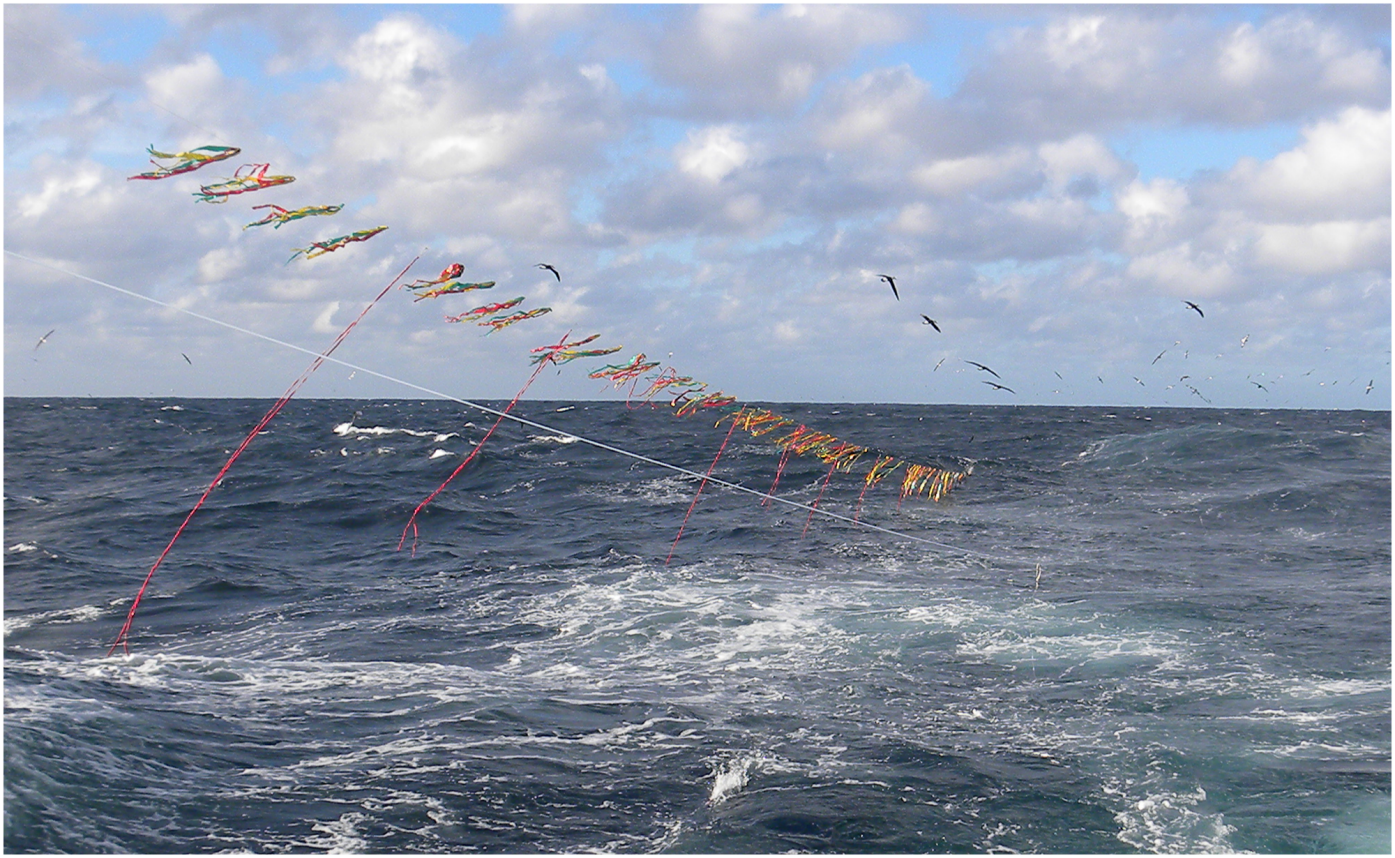
Image from the stern of a fishing vessel of the Uruguayan tori line deployed in situ showing the long and short streamers and the position to starboard in relation to the centrally deployed fishing mainline.

The tori line was designed based on the combination of other existing configurations such as those used by “Projeto Albatroz” [22] and the Japanese fleet. The tori line consisted of three sections: the aerial section, the connection section and the towed device (Fig 2 and S1 Fig). During phase 1 the designs of these sections were as follows: the aerial section (100m length, 2.6 kg) consisted of a backbone made of monofilament (polyamide 2.0mm, 3.46 g / m) with two types of streamers of different length and materials (long streamers and short streamers). The first streamer was positioned to fly 10m from the stern of the vessel. The backbone of the tori line was divided in three sections connected with small un-weighted swivels (size 4/0; 2.5 grs). The long streamers were red plastic bands or tubes (16 g/m), tied at their midpoint to the tori line backbone with a small un-weighted swivel (size 4/0, 2.5 g; S2a Fig). The tori line featured nine long pairs of streamers. The length of streamers decreased towards the towed device as follows: (1) 5.80m, (2) 5.00m, (3) 4.20m, (4) 3.70m, (5) 3.20m, (6) 2.80m, (7) 2.50m, (8) 2.00m and (9) 1,70m. In the absence of wind, long streamers touched the water surface. Long streamers were placed at a distance of 5m from each other, with the exception of those in positions 1 and 2, which were deployed 10m apart. The last pair of long streamers was placed at 55 m from the stern of the vessel. Short streamers consisted of three 2m nylon/plastic ribbons (1.9 g/m) of different colours (red, blue, yellow, green) tied at their midpoint to the backbone (S2b Fig). The short streamers were attached every meter between 15 and 55m from the stern, thus each long streamer was followed by four short streamers. After 55m short streamers were attached at 1 m intervals up to 75 m and thereafter spaced at 2 m intervals. At 65, 75, 85 and 95m a bunch of white streamers were attached to help demarcate distances to help estimate aerial coverage (see below). The connection section (20m length) was made out of monofilament (polyamide, 2.0mm) joined to the aerial section with an un-weighted swivel and to the towed device by a loop. Finally, the towed device consisted of a 30m multifilament (polyethylene 4.0–6.0 mm) line with 0.80m packing straps placed every 0.20m (approx.) and tied at their midpoint to the line. This line was attached to the connection section with a snap.

#### Experimental design and data collection

On each trip we employed two different longline set treatments based on a randomized order: sets with a tori line (tori line treatment) and sets without a tori line (control treatment). The null hypothesis that tori line use does not reduce the incidental bycatch of seabirds was tested. The capture of seabirds was the response variable. Tori line treatments involved the construction of a tori pole (one in each side of the vessel), a structure composed of two steel pipes forming a 90°angle adapted on each vessel to position the attachment point of the tori line at 6 m above sea level and 5m (range: 4-6m) to the side of the setting station. The pipe that attached the tori line was made by either a single piece or two pieces (one of smaller diameter inside the other); the latter allowed to move the attachment point to the desired distance from the setting station. The tori line was set on the leeward side of the mainline, according to the direction of the wind and the vessel course at the start of the set.

All seabird bycatch was sampled. For each set, data recorded included the number of bycaught birds, the species, how they were captured and whether they were retrieved dead or alive. Birds recovered alive were assumed to have been caught during hauling and were not included further in the analysis. Variables recorded at the beginning of the setting operation included time of the set, fishing effort (number of hooks), wind speed (Beaufort scale) and moon phase (i.e. new moon, first quarter, full moon and last quarter, following [15]). Sets starting in daylight were considered diurnal even though most finished in darkness); otherwise, sets were classified as nocturnal [17]. The randomised order of treatments resulted in 36% of the sets being diurnal (18% with each treatment) and the remaining 64% nocturnal (33% with the tori line treatment and 31% with the control treatment). Moon phases in nocturnal sets have a proven effect on seabird bycatch in this fishery [15, 17]. The percentage of sets with and without a tori line under each moon phase was similar (1% and 3% during a new moon, 17% and 18% during the first quarter, 12% and 9% during a full moon and 3% and 1% during the last quarter, for the tori line and control treatments, respectively). Wind may have an effect on seabird flight, and therefore on their abundance in the area, and also potential effects on tori line performance [17]. Considering wind speed, longline sets were classified in two categories (low wind, i.e. 0–2 and high wind i.e. 3–5, on the Beaufort scale). Forty five percent and 55% of the sets were conducted with low and high wind speeds, respectively, and in both cases a tori line was used in approximately half of them (22% and 23% with low wind and 29% and 26% with high wind, for the tori line and control treatments, respectively). Therefore, both treatments were exposed to similar conditions. Additionally, we attempted to control other relevant variables in the experimental design. These include the area and season, as the experiment was conducted in the area with the highest bycatch rate and during the peak season of seabird bycatch (see above). Bird abundance during setting was not assessed due to the impossibility of conducting bird counts during the night sets.

Tori line efficiency was also evaluated in accordance with two tori line attributes, the aerial coverage and the entanglement rate. The aerial coverage was measured by counting the number of long streamers (up to 55m) and short white streamers (every 10m thereafter) that remained out of the water. Because the aerial coverage had an ample variation due to the waves, we recorded its range and then the median value was used for analysis. For example, an aerial coverage that ranged between the first and the second bunch of white streamers, i.e. 65 -75m, was estimated as 70m.

### Phase 2. Reducing entanglement and rupture rates

The tori line was modified based on the observations made during Phase 1 (see Results). In order to minimise entanglement and ruptures in the aerial section, the diameter of the backbone was changed from 2.0mm (3.46 g / m) to 2.5mm (5.41 g / m). The 2.5 g swivels were replaced by larger swivels (12.5 g; S2c Fig) in line with the diameter of the new backbone. Two swivels were added in the distal third of the backbone to prevent it from becoming twisted. Because most entanglements involved the towed device, its length was reduced from 30m to 15m, and packing straps were placed every 0.5 m instead of every 0.2m. Additionally, ten packing straps of 2m long folded at the midpoint were placed at the end of the towed device. The diameter of the connection section was intentionally kept at 2.0mm to use this section as a weak link in case of entanglement.

In order to avoid entanglements between the tori line and the fishing gear (see Results), a system was devised to adjust the deployment of the tori line to leeward. The system was slightly different for the F/V and the R/V. In the F/V the tori line was attached to a rope that was passed through the rings of the tori poles on each side of the vessel (S3a and S3b Fig) and was shifted from the port side to the starboard and *vice versa* by pulling and fastening a lazy line. In the R/V, the tori line was attached to a pole located on the midline of the vessel and was shifted between the tori poles on each side by means of two lazy lines attached to the tori line via snaps (S3c Fig). In phase 2, setting operations were nocturnal with the exception of six sets (8.2%) that started in daylight in the F/V.

### Data analysis

The sample unit for all analyses was the longline sets. In phase 1, seabird bycatch was recorded in 20% of the longline fishing sets (n = 100 sets). Captures per set ranged between 1 and 3 individuals; the exceptions were two sets with 9 and 11 birds caught. The catch rate was firstly modeled with generalised linear models (GLMs) using the number of bird caught as a response variable and the logarithm of the fishing effort (in number of hooks) as an off set. A preliminary analysis with a Poisson distribution showed strong overdispersion; therefore, negative binomial GLMs with a log link function were fitted using the glm.nb function of MASS library (version 7.3–47) in R. The following categorical explanatory variables were considered: wind (low and high; see above), time of the set (day and night) and tori line. Additionally, the interaction between time of the set and tori line was included. The factor moon phase was excluded as its categories were highly unbalanced (see above). The same occurred with the used bait. Additionally, it was not possible to confirm whether the birds were caught with squid or fish in those sets where a mix of bait was used. Sets during one trip can be considered more similar (e.g. similar seabird assemblage, fishing area and operation) than those on other trips. Therefore, we alternatively fitted a Negative Binomial generalized linear mixed model (GLMM), with a log link function, adding ‘‘fishing trip” as a random factor. We used the glmer.nb function, lme4 library (version 1.1–13) [23] to fit this model. In order to compare rupture rates of the tori line between phase 1 and 2, a GLMM with a logit link function, assuming a binomial distribution and using ‘‘fishing trip” as a random factor was fitted with the function glmer, lme4 library; the categorical factors “phase” (phases 1 and 2), side of the vessel (“side”) from where the tori line was deployed (starboard and port side) and wind (low and high; see above), and their interactions, were included as explanatory variables. Data from phase 1 only included those sets with a tori line. For all the analyses, a likelihood Ratio Test (LRT) was used to test the significance of each covariate; single terms were dropped and the resulting sub-model compared with the full model. Sequential deletions of non-significant terms were conducted until only significant covariates remained in the model. The model fit of the GLM and GLMM from phase 1 were compared using the Akaike information criteria (AIC). For each significant categorical variable from the GLMM of phase 2, the rate of change in the odds was calculated as the exponent of the parameter estimate. This is a measure of the change of the tori line breaking under one condition (e.g. with low wind) compared with the change of the tori line breaking under another condition (e.g. high wind). The 95% confidence limits (c.l.) were calculated using the exponent of the parameter plus or minus 1.96 times the standard error and presented as a percentage. Comparison between the aerial coverage of the tori line in phases 1 and 2 were conducted using a Mann–Whitney test. The aerial coverage of the tori line is presented as average ± 1 standard deviation (SD). Significance was set at p<0.05.

## Results

### Effectiveness of the tori line

One hundred sets (51 and 49 with and without a tori line, respectively) were made and 102,984 hooks (52,371 and 50,613 hooks, respectively) were deployed during phase 1. Fifty birds were incidentally caught (0.49 birds/1,000 hooks), of which 43 birds were captured on sets without a tori line (BCPUE = 0.85 birds/1,000 hooks) and 7 birds on those with a tori line (BCPUE = 0.13 birds/1,000 hooks) (Table 1). The negative binomial GLM with all covariates indicated that only the tori line affects catch rates; after applying single term deletions from the full model and the Likelihood Ratio Test (LRT), only the factor tori line was a significant predictor of bycatch rate (LRT, χ2 = 8.71, degrees of freedom (df) = 1, p = 0.003). The estimated coefficient indicated that the use of tori line significantly reduced seabird bycatch (Table 2). After removing the remaining non-significant covariates, the model including tori line was significantly better than the model constructed without this term (LRT, χ2 = 10.82, degrees of freedom (df) = 1, p = 0.001) and its coefficient again showed the effectiveness of this mitigation measure to reduce seabird bycatch rates (Table 2). This model showed a relatively good fit, with no indication of over-dispersion (Table 2). The model with the inclusion of the fishing trip as a random factor showed a similar result, but did not improve the model fit based in the AIC value (AIC = 171.8, see Table 2 for AIC values of the GLMs models), and therefore was excluded.

**Table 1:** Number of birds incidentally captured in each treatment during Phases 1 and 2. The observed fishing effort was as follows: Phase 1: with tori line = 51 sets and 52,371 hooks, without a tori line = 49 sets and 50,613 hooks; Phase 2: with tori line = 73 sets and 47,554 hooks.

Species	Phase 1			Phase 2
	With tori line	Without tori line	Total	With tori line
Southern Royal Albatross *Diomedea epomophora*	0	4	4	1
Northern Royal Albatross *Diomedea sanfordi*	2	1	3	0
White-capped Albatross *Thalassarche steadi*	0	2	2	0
shy-type Albatross *Thalassarche cauta/steadi*	0	0	0	1
Black-bowed Albatross *Thalassarche melanophris*	3	26	29	11
Southern Giant Petrel *Macronectes giganteus*	0	1	1	0
White-chinned Petrel *Procellaria aequinoctialis*	2	8	10	3
Great Shearwater *Ardenna gravis*	0	1	1	0
**Totals**	7	43	50	16

**Table 2:** Estimated coefficients and standard errors (SE) of the GLMs (Negative Binomial) for the bycatch rate of seabirds. The Akaike information criteria (AIC) values and the dispersion parameters are shown for each model. All models were fitted using the log link function. Note that for every factor, one category is fixed (intercept), which serves as the standard for comparisons with other levels.

Model	Factors	Coefficient	SE	z	p	AIC	Dispersion
***Wind + TimeSet + Tori Line + TimeSet*: *Tori Line + offset (LogEffort)***						173.5	**1.224**
(Intercept)		-5.87	0.67	-8.72	<0.00001		
Wind_high		-0.71	0.59	-1.21	0.22780		
TimeSet_night		-1.03	0.74	-1.40	0.16260		
Toriline_use		-2.41	0.98	-2.46	0.01370		
TimeSet_night:Toriline_use		0.86	1.24	0.69	0.48740		
***Tori Line + offset (LogEffort)***						171.3	**1.229**
(Intercept)		-6.65	0.38	-17.53	<0.00001		
Toriline_use		-2.15	0.63	-3.39	0.00071		

Three of the birds captured during the tori line treatment occurred on sets where the tori line became entangled and as result had a reduced aerial coverage. Of the remaining birds caught on this treatment, two were captured after part or all of the tori line was lost following a rupture caused by tension produced by either the waves or wind (see below). The catch rate of birds on sets without ruptures of the tori line was 0.07 birds/1000 hooks and on those with entanglements or ruptures was 0.21 birds/1000 hooks.

Tori line ruptures were recorded on 47% of the longline sets deployed under this treatment. In 20 sets the tori line became entangled with the fishing gear and broke immediately. In four sets the tori line broke due to the high tension caused by the effects of wind and waves. The average aerial coverage of the tori line was 73.3 ± 12.3 m and 46.8 = ± 9.4 m before and after rupture, respectively. The details of the entanglements could only be recorded in eight sets. Six of those entanglements involved buoys and two were with radio buoys. Two occurred following a change in vessel course or wind direction, which caused the tori line to move windward of the fishing gear. Seven of the entanglements were associated with the towed device and ruptures occurred either along the extension of this device or in the aerial part of the tori line. All ruptures caused by wind and waves were in the aerial section.

### Reducing rupture rates

During phase 2, a total of 73 longline sets (47,554 hooks) with a tori line were deployed. Seven (9.6%) rupture events were recorded; six occurred after entanglement with buoys, and one was caused by entanglement with a hook. The binomial GLMM showed that there was a significant decrease in the rupture rate in comparison with phase 1 (Table 3). The rate of change in odds showed that the chance of the tori line breaking in phase 2 was 4% (95% c.l. = 1–18%) of that during phase 1. The wind-by-phase and side of the vessel-by-phase interactions were not significant, and were excluded from the model. The factors “wind” and “side of the vessel” were significant (Table 3). The chance of the tori line breaking when deployed to starboard was over five times higher than when deployed to the port side (Table 3). There was a significant increase in the rupture rate during higher wind speeds; the chance of the tori line becoming entangled in low winds was 14% of that during high winds (Table 3).

**Table 3:** Estimated coefficients and standard errors (SE) of the GLMM (Binomial) for the ruptures of the tori line. The rate of change in the odds and 95% confidence limits (c.l.) is presented for each variable. Variance of random effect “fishing trip” was 0.33 and standard deviation was 0.57.

Fixed effects	Coefficient	SE	z	p	Rate of change in odds	95% c.l.
(Intercept)		0.55	-0.34	0.73693		
Phase_2	-3.25	0.78	-4.15	0.00003	4	1–18
Side_Stardboard	1.73	0.72	2.41	0.01580	566	137–2347
Wind_low	-1.99	0.67	-2.98	0.00291	14	4–51

**Fig 3:**
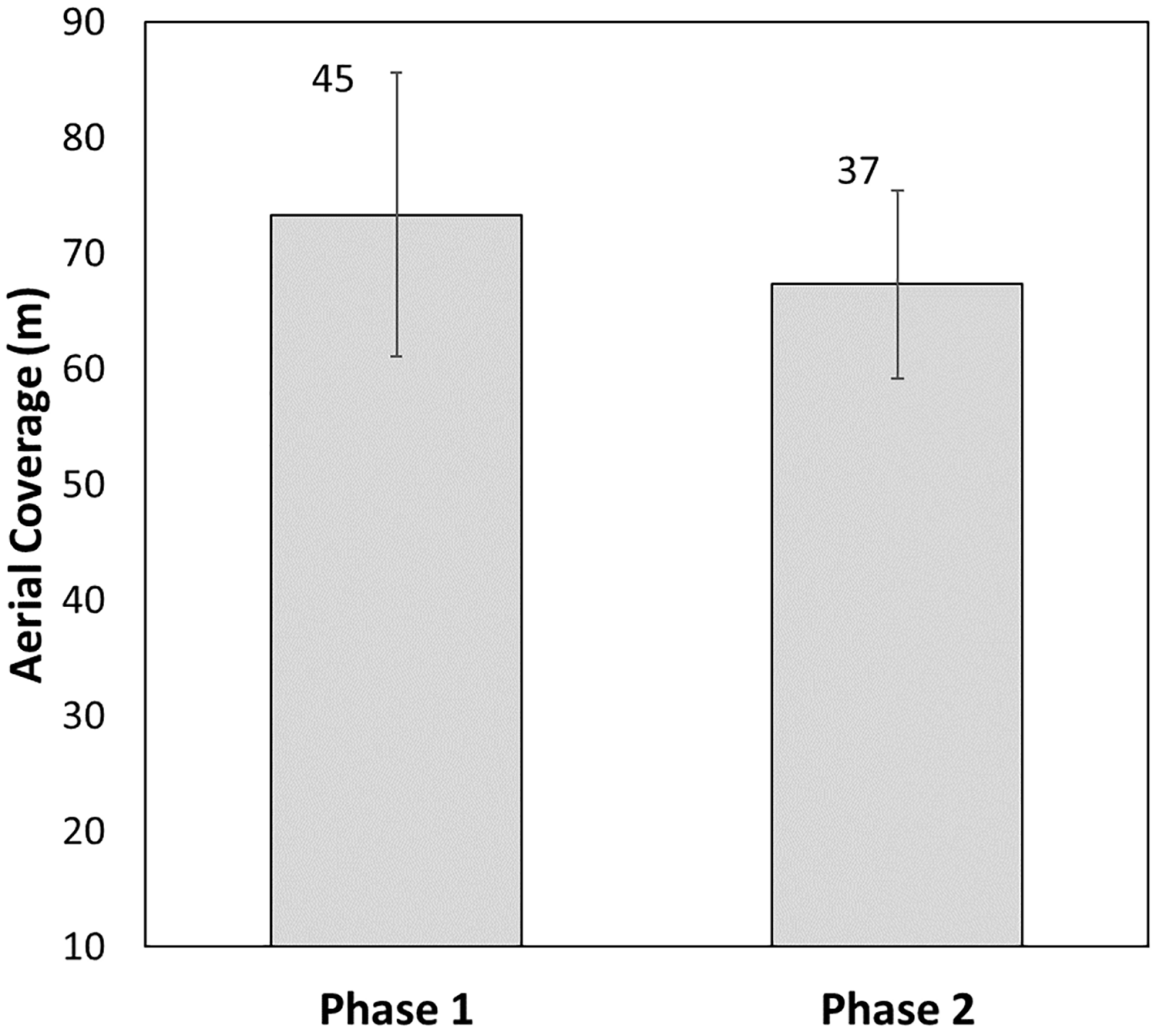
Mean aerial coverage of the tori line in phases 1 and 2. Error bars represent ± 1 standard deviation (SD). The number above each bar indicates the sample size.

The aerial coverage of the tori line in phase 2 was 67.3m ± 8.2m, significantly lower than in phase 1 (Mann–Whitney, p = 0.014, n = 82; Fig 3). During phase 2, the tori line was successfully shifted to the opposite (leeward) side of the vessel in seven of a total of eight longline sets. The one set where the tori line became entangled was caused by a buoy and resulted in a rupture during the maneuver.

Sixteen seabirds (0.34 birds/1000 hooks) were incidentally caught in phase 2 (Table 1). One of these captures occurred after the tori line broke.

## Discussion

The results of our experimental study to reduce seabird bycatch in pelagic longline fisheries demonstrate the effectiveness of a tori line over a control treatment. Currently, recommended best-practices to mitigate seabird mortality in pelagic longline fisheries involve the combined use of night setting, tori lines and specific weighting regimes in branch lines [3, 6, 24]. In the south Atlantic Ocean, where this research was carried out, the recommendation 11–09 (http://www.iccat.int/en/Recs-Regs.asp) of the International Commission for the Conservation of Atlantic Tunas (ICCAT) stipulates that south of 25°S, ICCAT members shall ensure that all longline vessels with lengths of 20m and over use at least two of these mitigation measures. The present study provides evidence for first time that this mitigation measure can be used in a range of medium size pelagic longline vessels (~25-40m). In vessels smaller than 25m, and especially those <20m, the applicability of a tori line should be assessed. In our study the tori line was towed from a height of 6m above sea level and a horizontal distance of 5m (range 4-6m) from the setting station. However, this configuration may be difficult to achieve on smaller vessels, especially those with narrow beams.

**Fig 4:**
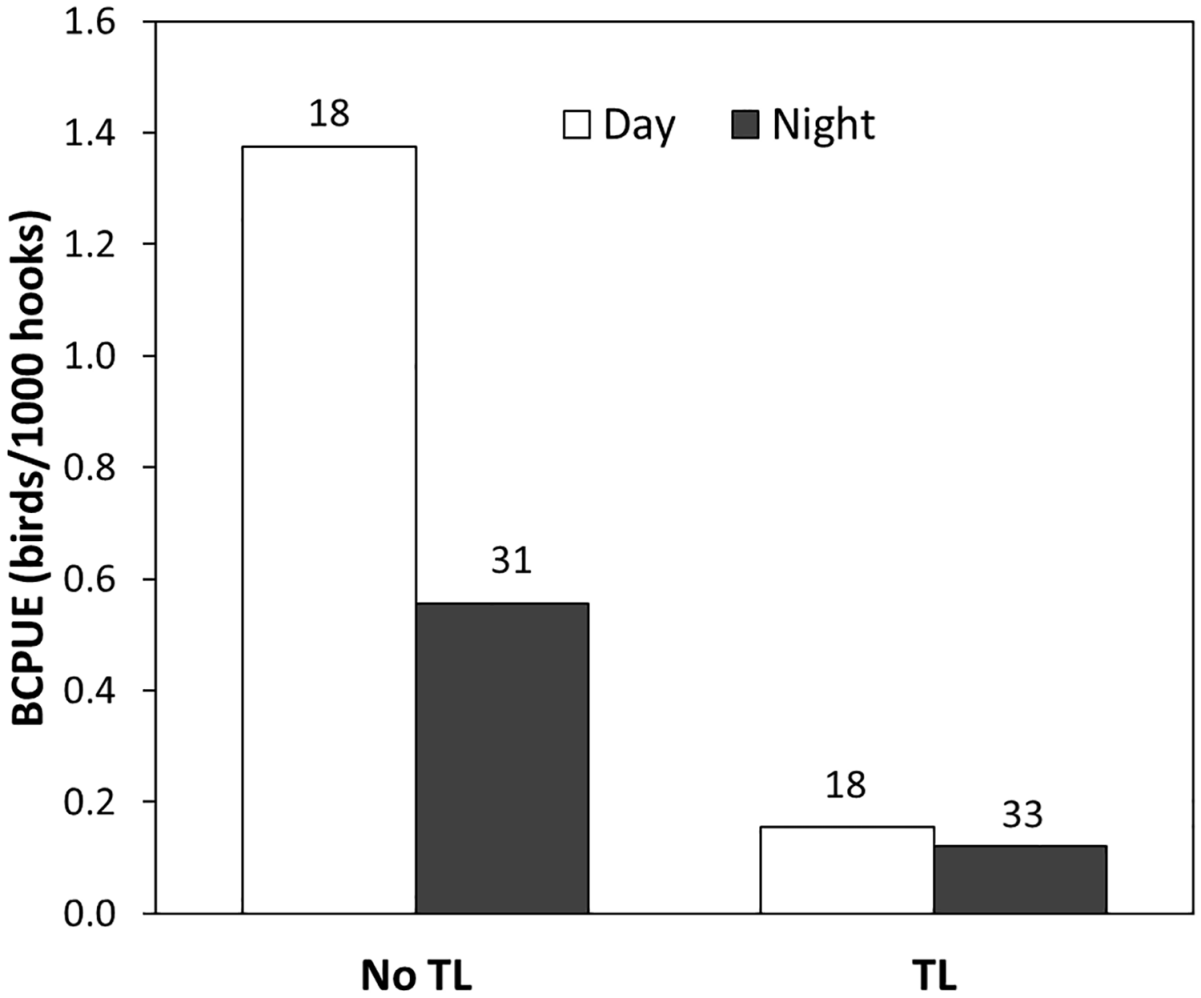
Bird capture per unit of effort (BCPUE; birds/1000 hooks) during day and night longline sets without tori line (No TL) and with tori line (TL). The number above each bar indicates the sample size.

The results of our study showed that the bycatch rates did not vary between longline sets deployed with a tori line as a single mitigation measure and those with the combined use of a tori line and night setting (Fig 4, and see Table 2; the non-significant interaction between these factors). This is likely to be related to moon phase, as 29 of the 33 nocturnal sets deployed with a tori line were in the first quarter and full moon phases (see Materials and methods) rendering night setting less effective at reducing seabird bycatch [15, 17]. Additionally, two of the four birds captured during these sets occurred on a set where the tori line had become entangled and resulted in a reduced aerial coverage.

The high rupture rate of the tori line caused either by entanglements or tension recorded in phase 1 of our research was significantly reduced in phase 2 (4%, 95% c.l. = 1–18%) following modification of the tori line design and deployment. The rupture rates were significantly higher when the tori line was deployed from the starboard side and under higher wind speeds. This is because buoys (foam bullet and rigid buoys), responsible for most entanglements, are also set from the starboard side of the vessel. In bad weather, ruptures can be caused either by high tension in the backbone or by entanglements with fishing gear due to the effect of large waves and high speed winds. However, we were able to demonstrate that the tori line should not always be deployed from the same side of the vessel, as when it is deployed to windward it can drift across the fishing gear, increasing the chance of entanglements. In phase 2, through design modifications we succeeded in shifting the tori line from one side of the vessel to the other when changes in vessel or wind direction left the tori line windward, thus eliminating this type of entanglement. The one exception was due to a lack of coordination between the fishing master and the deck crew. Our field observations suggest that when the tori line is positioned leeward on the starboard side, the buoys should be deployed toward the midline of the vessel wash and if possible toward the port side instead. Further studies in medium size pelagic longline vessels should focus on reducing entanglements between the tori line and buoys.

The reduction in length of the towed device (i.e. multifilament line with packing straps) is also likely to have contributed to reduced entanglement rates and ruptures in phase 2. However, this also significantly reduced the aerial coverage of the tori line by 6m on average (see Fig 2). This suggests that further modifications in the drag section may be required to maintain efficacy whilst minimising entanglements. During phase 2 the catch rate of seabirds (0.34 birds/1000 hooks) was lower than that recorded on the control treatment in 2009–2011 (0.85 birds/1,000 hooks), but was higher than the catch rate observed with a tori line during the same period (0.13 birds/1,000 hooks). These differences are not easy to understand due to the lack of control sets (i.e. without tori line) during phase 2. In each of the two phases the vessels operated in approximately the same area (Fig 1), and during the period of peak seabird bycatch (May-November)[15] with the exception of a few sets in March and December during phase 2. The reduction in the tori line aerial coverage may have affected this (see below). However, it is also important to mention that all sets with seabird captures, except one, took place in 2012. Half of the total captures occurred on the few diurnal sets (see Materials and methods) recorded that year, and another occurred after an entanglement when the tori line broke and aerial coverage was reduced as a result. However, a greater proportion of diurnal sets with a tori line were recorded in phase 1. Similarly, the proportion of night sets with first quarter and full moon phases were greater (see above) in phase 1 than phases 2 (25 of the 67 nocturnal sets). An inter-annual variation in the seabird abundance may explain the increased bycatch rate.

The aerial coverage of the tori line (~70m) is enough to protect the distance astern (≤ 50m) where the highest seabird attack rates occur in the absence of mitigation measures, as previously identified in this fishery [25]. The tori line is known to displace seabirds further away from the vessel [9, 11] and therefore a better aerial coverage of the tori line is always desirable, especially when baited hooks are still at depths accessible to diving seabirds beyond its protection. Preliminary data recorded in this fishery in 2011–2015 (including fishing trips from this study) showed that the baited hooks on standard branch line configurations (a 75g weighted swivel attached 4.5 m from the hook) achieved an average sink rate of 0.15 ms^–1^ (standard deviation, SD = 0.06 ms^–1^, n = 112 hooks) in the upper 2 m (DINARA unpublished data). This indicates that, on average, at the end of the aerial coverage of the tori line the baited hooks are located at a depth of 2.4m (assuming a mean setting speed of 8.1 knots, see Materials and methods), where they are still available to almost all species of albatrosses and petrels [26–29]. However, the use of branch lines with a weight of 65g attached 1m from the hook achieved an average sink rate of 0.28ms^-1^ (SD = 0.10 ms^-1^, n = 106 hooks) in the upper 4m (DINARA unpublished data), indicating that, on average, beyond the tori line protection baited hooks are at the maximum diving depth of albatrosses (~4.5m)[28] or deeper. However, hooks would still be accessible to petrels, and thus to albatrosses which gain access to baits through secondary interactions with petrels [25]. The branch line configuration with weights placed at 1 m from the hook could further reduce seabird bycatch and complement the tori line performance. Therefore, as mentioned above, the tori line needs to be complemented with the use of other mitigation measures, of which night setting [8, 15, 30] and the use of appropriate branch line weighting [6, 12, 31] have the greatest scientific support in reducing seabird mortality. This tori line design has proved efficient at reducing seabird bycatch in an area that has records of very high bycatch rates at global level. Accordingly, it is also potentially effective at reducing seabird bycatch on other medium size vessels fishing in the southern hemisphere. Further trials with this tori line to improve its design, effectiveness and applicability to multiple vessels should be conducted.

## Supporting information

S1 Table Dataset. Details for phase 1 and 2.
(PDF)

S2 Table Dataset for effectiveness experiment. (PDF)

S3 Table Dataset for the analysis of entanglements and ruptures. (PDF)

S1 Fig Schematic representation of the tori line. The tori line consisted of three sections: the aerial section (100m), the connection section (20m) and the towed device (30 m in phase 1 and 15 m in phase 2).
(TIF)

S2 Fig Pictures showing the attachments of (a) long streamers, (b) short streamers and (c) swivels to the backbone. (TIF)

S3 Fig Schematic representation of the stern of the vessel and the system used to shift the tori line from one side of the vessel to the other depending on wind direction in phase 2. In the F/V the tori line was attached to a rope that was passed through the rings of the tori poles on each side of the vessel (S3a and S3b) and was shifted from the port side to the starboard and vice versa by pulling and fastening a lazy line. In the R/V, the tori line was attached to a pole located on the midline of the vessel and was shifted between the tori poles on each side by means of two lazy lines attached to the tori line via snaps (c).
(TIF)
