# Supplementary material for: Effectiveness of tori line use to reduce seabird bycatch in pelagic longline fishing
Source: PLoS One. 2017 Sep 8;12(9):e0184465. doi: 10.1371/journal.pone.0184465 (PMC5590930; doi:10.1371/journal.pone.0184465)
Supplement: S1 Table — Details for phase 1 and 2. (PDF) [file pone.0184465.s001.pdf]

Table S1. Dataset. Details for phase 1 and 2.

Phase	Year	Trip	#_Sets	#_Hooks	#_SetswithoutToriline	#_HookwithoutToriline	#_birdswithoutToriline	#_SetswithToriline	#_HookswithToriline	#_birdswithToriline	#_ruptures
1	2009	1	10	12834	5	6324	4	5	6510	0	2
1	2009	2	6	2430	3	1260	1	3	1170	0	1
1	2009	3	7	8345	3	3415	0	4	4930	0	2
1	2010	4	4	4170	2	2180	0	2	1990	0	0
1	2010	5	9	8910	4	3960	0	5	4950	0	4
1	2010	6	8	3200	4	1600	11	4	1600	0	3
1	2010	7	5	5660	2	2020	1	3	3640	0	1
1	2010	8	2	2100	1	1250	8	1	850	0	1
1	2011	9	14	19716	7	9960	5	7	9756	1	0
1	2011	10	7	9800	4	5800	4	3	4000	1	1
1	2011	11	13	5769	7	3104	9	6	2665	3	3
1	2011	12	7	8850	3	4090	0	4	4760	1	3
1	2011	13	8	11200	4	5650	0	4	5550	1	3
2	2012	14	4	5584	-	-	-	4	5584	4	0
2	2012	15	9	11250	-	-	-	9	11250	5	1
2	2012	16	5	5970	-	-	-	5	5970	1	1
2	2012	17	8	3600	-	-	-	8	3600	4	0
2	2013	18	9	4050	-	-	-	9	4050	0	1
2	2013	19	9	4050	-	-	-	9	4050	0	2
2	2014	20	13	5850	-	-	-	13	5850	0	0
2	2015	21	5	2250	-	-	-	5	2250	0	0
2	2016	22	11	4950	-	-	-	11	4950	2	2

Notes: Phase: Experimental phases of this research (see Methods); Trip = Number id of the trip; #_Sets = total number of longline sets; #_Hooks = total number of hooks; #_SetswithoutToriline = total number of longline sets without tori line; #_HookwithoutToriline = total number of hooks without tori line; #_birdswithoutToriline = total number of birds captured without tori line; #_SetswithToriline = total number of longline sets with tori line; #_HookswithToriline = total number of hooks with tori line; #_birdswithToriline = total number of birds captured with tori line; #_ruptures = total number of longline sets with ruptures of tori line.
