# Supplementary material for: Effectiveness of tori line use to reduce seabird bycatch in pelagic longline fishing
Source: PLoS One. 2017 Sep 8;12(9):e0184465. doi: 10.1371/journal.pone.0184465 (PMC5590930; doi:10.1371/journal.pone.0184465)
Supplement: S2 Table — (PDF) [file pone.0184465.s002.pdf]

Table S2. Dataset for Effectiveness experiment.

Trip	Effort	Beaufort	Wind	TimeSet	Toriline	Birds
1	1302	4	2	1	0	0
1	1302	2	1	1	1	0
1	1302	3	2	2	0	0
1	1302	2	1	1	1	0
1	1302	2	1	2	1	0
1	1302	2	1	2	1	0
1	1302	2	1	2	1	0
1	1302	3	2	2	0	0
1	1302	3	2	1	0	1
1	1116	4	2	2	0	3
2	450	3	2	1	1	0
2	450	2	1	2	0	0
2	450	2	1	1	0	0
2	360	3	2	2	1	0
2	360	2	1	1	0	1
2	360	1	1	2	1	0
3	1190	2	1	2	0	0
3	1360	0	1	2	1	0
3	960	3	2	2	0	0
3	1020	5	2	2	1	0
3	1360	3	2	1	1	0
3	1265	5	2	1	0	0
3	1190	1	1	2	1	0
4	980	3	2	2	0	0
4	1030	3	2	2	1	0
4	960	4	2	2	1	0
4	1200	3	2	2	0	0
5	990	4	2	2	1	0
5	990	6	2	2	0	0
5	990	3	2	2	1	0
5	990	3	2	2	0	0
5	990	3	2	2	1	0
5	990	3	2	2	0	0
5	990	2	1	2	1	0
5	990	2	1	2	1	0

5	990	3	2	2	0	0
6	400	2	1	1	0	11
6	400	2	1	1	1	0
6	400	1	1	1	1	0
6	400	2	1	1	0	0
6	400	4	2	1	1	0
6	400	2	1	1	0	0
6	400	1	1	1	0	0
6	400	3	2	1	1	0
7	1000	1	1	1	1	0
7	700	4	2	1	0	1
7	1320	1	1	1	1	0
7	1320	2	1	2	0	0
7	1320	3	2	2	1	0
8	1250	5	2	1	0	8
8	850	5	2	2	1	0
9	1149	4	2	2	0	3
9	1467	3	2	2	1	0
9	1476	0	1	2	1	0
9	1485	2	1	2	0	0
9	1485	3	2	2	0	0
9	1449	0	1	2	0	2
9	1485	0	1	2	1	1
9	1359	3	2	2	1	0
9	1449	1	1	2	1	0
9	1449	2	1	2	0	0
9	1494	4	2	2	0	0
9	1485	2	1	2	1	0
9	1449	4	2	2	0	0
9	1035	3	2	2	1	0
10	1200	2	1	2	1	0
10	1530	3	2	1	0	3
10	1400	1	1	1	1	0
10	1400	1	1	2	0	0
10	1400	4	2	1	1	1
10	1400	2	1	2	0	0
10	1470	2	1	2	0	1
11	450	2	1	2	1	1
11	450	2	1	2	0	3

11	450	2	1	2	0	2
11	450	1	1	2	1	0
11	450	1	1	2	0	2
11	450	3	2	2	1	2
11	436	0	1	2	0	0
11	439	1	1	2	0	2
11	440	3	2	2	1	0
11	437	3	2	2	1	0
11	438	2	1	2	0	0
11	438	3	2	2	1	0
11	441	2	1	2	0	0
12	800	6	2	2	1	0
12	1450	4	2	1	0	0
12	1320	3	2	1	1	1
12	1320	4	2	1	0	0
12	1320	4	2	1	1	0
12	1320	6	2	1	1	0
12	1320	4	2	1	0	0
13	1300	4	2	2	0	0
13	1550	3	2	1	0	0
13	1450	4	2	1	1	1
13	1350	5	2	1	0	0
13	1300	5	2	1	1	0
13	1300	5	2	2	1	0
13	1450	1	1	1	0	0
13	1500	4	2	1	1	0

Notes: Each row correspond to a single longline set (sample unit). Trip = Number id of the trip; Effort = total number of hooks; Beaufort = Beaufort scale; wind, categories based on Beaufort scale (see Methods), 1 = low, 2 = high; TimeSet = time of the longline set, 1 = day, 2 = night; Toriline, 0 = without toriline, 1 = with tori line; Birds = total number of birds captured.

.
