# Supplementary material for: Effectiveness of tori line use to reduce seabird bycatch in pelagic longline fishing
Source: PLoS One. 2017 Sep 8;12(9):e0184465. doi: 10.1371/journal.pone.0184465 (PMC5590930; doi:10.1371/journal.pone.0184465)
Supplement: S3 Table — (PDF) [file pone.0184465.s003.pdf]

**Table S3. Dataset for the analysis of entanglements and ruptures.**

| Phase | Trip | Toriline | Side | ruptures | Beaufort | Wind |
|---|---|---|---|---|---|---|
| 1 | 1 | 1 | starboard | 0 | 2 | 1 |
| 1 | 1 | 1 | starboard | 0 | 2 | 1 |
| 1 | 1 | 1 | starboard | 1 | 2 | 1 |
| 1 | 1 | 1 | starboard | 1 | 2 | 1 |
| 1 | 1 | 1 | starboard | 0 | 2 | 1 |
| 1 | 2 | 1 | portside | 0 | 3 | 2 |
| 1 | 2 | 1 | starboard | 1 | 3 | 2 |
| 1 | 2 | 1 | portside | 0 | 1 | 1 |
| 1 | 3 | 1 | portside | 0 | 0 | 1 |
| 1 | 3 | 1 | portside | 1 | 5 | 2 |
| 1 | 3 | 1 | portside | 1 | 3 | 2 |
| 1 | 3 | 1 | portside | 0 | 1 | 1 |
| 1 | 4 | 1 | portside | 0 | 3 | 2 |
| 1 | 4 | 1 | portside | 0 | 4 | 2 |
| 1 | 5 | 1 | starboard | 1 | 4 | 2 |
| 1 | 5 | 1 | starboard | 1 | 3 | 2 |
| 1 | 5 | 1 | starboard | 1 | 3 | 2 |
| 1 | 5 | 1 | starboard | 0 | 2 | 1 |
| 1 | 5 | 1 | starboard | 1 | 2 | 1 |
| 1 | 6 | 1 | portside | 1 | 2 | 1 |
| 1 | 6 | 1 | portside | 1 | 1 | 1 |
| 1 | 6 | 1 | portside | 0 | 4 | 2 |
| 1 | 6 | 1 | portside | 1 | 3 | 2 |
| 1 | 7 | 1 | starboard | 0 | 1 | 1 |
| 1 | 7 | 1 | portside | 0 | 1 | 1 |
| 1 | 7 | 1 | starboard | 1 | 3 | 2 |
| 1 | 8 | 1 | starboard | 1 | 5 | 2 |
| 1 | 9 | 1 | starboard | 0 | 3 | 2 |
| 1 | 9 | 1 | starboard | 0 | 0 | 1 |
| 1 | 9 | 1 | starboard | 0 | 0 | 1 |
| 1 | 9 | 1 | portside | 0 | 3 | 2 |
| 1 | 9 | 1 | portside | 0 | 1 | 1 |
| 1 | 9 | 1 | portside | 0 | 2 | 1 |
| 1 | 9 | 1 | portside | 0 | 3 | 2 |

| | | | | | | |
|---|---|---|---|---|---|---|
| 1 | 10 | 1 | portside | 0 | 2 | 1 |
| 1 | 10 | 1 | portside | 0 | 1 | 1 |
| 1 | 10 | 1 | starboard | 1 | 4 | 2 |
| 1 | 11 | 1 | starboard | 0 | 2 | 1 |
| 1 | 11 | 1 | portside | 0 | 1 | 1 |
| 1 | 11 | 1 | starboard | 1 | 3 | 2 |
| 1 | 11 | 1 | starboard | 1 | 3 | 2 |
| 1 | 11 | 1 | portside | 1 | 3 | 2 |
| 1 | 11 | 1 | starboard | 0 | 3 | 2 |
| 1 | 12 | 1 | portside | 0 | 6 | 2 |
| 1 | 12 | 1 | portside | 1 | 3 | 2 |
| 1 | 12 | 1 | starboard | 1 | 4 | 2 |
| 1 | 12 | 1 | portside | 1 | 6 | 2 |
| 1 | 13 | 1 | starboard | 1 | 4 | 2 |
| 1 | 13 | 1 | portside | 0 | 3 | 2 |
| 1 | 13 | 1 | starboard | 1 | 5 | 2 |
| 1 | 13 | 1 | starboard | 1 | 4 | 2 |
| 2 | 14 | 1 | starboard | 0 | 3 | 2 |
| 2 | 14 | 1 | starboard | 0 | 6 | 2 |
| 2 | 14 | 1 | starboard | 0 | 3 | 2 |
| 2 | 14 | 1 | portside | 0 | 3 | 2 |
| 2 | 15 | 1 | portside | 0 | 4 | 2 |
| 2 | 15 | 1 | starboard | 1 | 4 | 2 |
| 2 | 15 | 1 | starboard | 0 | 3 | 2 |
| 2 | 15 | 1 | starboard | 0 | 4 | 2 |
| 2 | 15 | 1 | portside | 0 | 2 | 1 |
| 2 | 15 | 1 | starboard | 0 | 5 | 2 |
| 2 | 15 | 1 | portside | 0 | 3 | 2 |
| 2 | 15 | 1 | starboard | 0 | 6 | 2 |
| 2 | 15 | 1 | starboard | 0 | 4 | 2 |
| 2 | 16 | 1 | starboard | 0 | 3 | 2 |
| 2 | 16 | 1 | starboard | 0 | 2 | 1 |
| 2 | 16 | 1 | starboard | 1 | 3 | 2 |
| 2 | 16 | 1 | starboard | 0 | 3 | 2 |
| 2 | 16 | 1 | starboard | 0 | 2 | 1 |
| 2 | 17 | 1 | starboard | 0 | 2 | 1 |
| 2 | 17 | 1 | starboard | 0 | 0 | 1 |
| 2 | 17 | 1 | starboard | 0 | 0 | 1 |
| 2 | 17 | 1 | starboard | 0 | 3 | 2 |

| | | | | | | |
|---|---|---|---|---|---|---|
| 2 | 17 | 1 | starboard | 0 | 1 | 1 |
| 2 | 17 | 1 | starboard | 0 | 4 | 2 |
| 2 | 17 | 1 | starboard | 0 | 3 | 2 |
| 2 | 17 | 1 | portside | 0 | 2 | 1 |
| 2 | 18 | 1 | starboard | 0 | 3 | 2 |
| 2 | 18 | 1 | starboard | 0 | 5 | 2 |
| 2 | 18 | 1 | starboard | 0 | 4 | 2 |
| 2 | 18 | 1 | starboard | 1 | 4 | 2 |
| 2 | 18 | 1 | portside | 0 | 2 | 1 |
| 2 | 18 | 1 | starboard | 0 | 0 | 1 |
| 2 | 18 | 1 | starboard | 0 | 1 | 1 |
| 2 | 18 | 1 | starboard | 0 | 3 | 2 |
| 2 | 18 | 1 | starboard | 0 | 2 | 1 |
| 2 | 19 | 1 | starboard | 0 | 6 | 2 |
| 2 | 19 | 1 | starboard | 0 | 3 | 2 |
| 2 | 19 | 1 | starboard | 0 | 3 | 2 |
| 2 | 19 | 1 | starboard | 1 | 3 | 2 |
| 2 | 19 | 1 | starboard | 0 | 2 | 1 |
| 2 | 19 | 1 | starboard | 1 | 5 | 2 |
| 2 | 19 | 1 | starboard | 0 | 3 | 2 |
| 2 | 19 | 1 | starboard | 0 | 2 | 1 |
| 2 | 19 | 1 | starboard | 0 | 0 | 1 |
| 2 | 20 | 1 | starboard | 0 | 3 | 2 |
| 2 | 20 | 1 | starboard | 0 | 3 | 2 |
| 2 | 20 | 1 | portside | 0 | 1 | 1 |
| 2 | 20 | 1 | starboard | 0 | 4 | 2 |
| 2 | 20 | 1 | starboard | 0 | 3 | 2 |
| 2 | 20 | 1 | starboard | 0 | 4 | 2 |
| 2 | 20 | 1 | starboard | 0 | 2 | 1 |
| 2 | 20 | 1 | portside | 0 | 1 | 1 |
| 2 | 20 | 1 | starboard | 0 | 3 | 2 |
| 2 | 20 | 1 | portside | 0 | 2 | 1 |
| 2 | 20 | 1 | portside | 0 | 3 | 2 |
| 2 | 20 | 1 | portside | 0 | 3 | 2 |
| 2 | 20 | 1 | portside | 0 | 5 | 2 |
| 2 | 21 | 1 | portside | 0 | 1 | 1 |
| 2 | 21 | 1 | portside | 0 | 2 | 1 |
| 2 | 21 | 1 | starboard | 0 | 3 | 2 |
| 2 | 21 | 1 | starboard | 0 | 2 | 1 |

| | | | | | | |
|---|---|---|---|---|---|---|
| 2 | 21 | 1 | starboard | 0 | 3 | 2 |
| 2 | 22 | 1 | starboard | 0 | 2 | 1 |
| 2 | 22 | 1 | starboard | 0 | 3 | 2 |
| 2 | 22 | 1 | starboard | 0 | 3 | 2 |
| 2 | 22 | 1 | portside | 0 | 3 | 2 |
| 2 | 22 | 1 | portside | 0 | 2 | 1 |
| 2 | 22 | 1 | starboard | 0 | 2 | 1 |
| 2 | 22 | 1 | portside | 0 | 2 | 1 |
| 2 | 22 | 1 | portside | 0 | 2 | 1 |
| 2 | 22 | 1 | starboard | 1 | 3 | 2 |
| 2 | 22 | 1 | starboard | 1 | 2 | 1 |
| 2 | 22 | 1 | portside | 0 | 1 | 1 |

Notes: Each row correspond to a single longline set (sample unit). Phase: Experimental phases of this research (see Methods); Trip = Number id of the trip; Toriline, 0 = without toriline, 1 = with tori line; side = vessel's side; ruptures, 0 = no, 1 = yes; Beaufort = Beaufort scale; wind, categories based on Beaufort scale (see Methods), 1 = low, 2 = high.
