# Supplementary figures and images for: Effectiveness of tori line use to reduce seabird bycatch in pelagic longline fishing
Source: PLoS One. 2017 Sep 8;12(9):e0184465. doi: 10.1371/journal.pone.0184465 (PMC5590930; doi:10.1371/journal.pone.0184465)

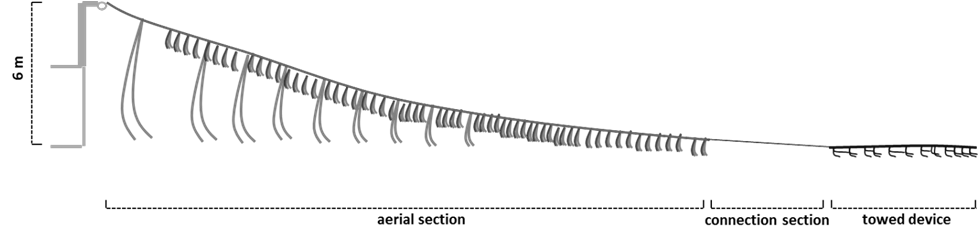

Supplement: S1 Fig — The tori line consisted of three sections: the aerial section (100m), the connection section (20m) and the towed device (30 m in phase 1 and 15 m in phase 2). (TIF) [file pone.0184465.s004.tif]

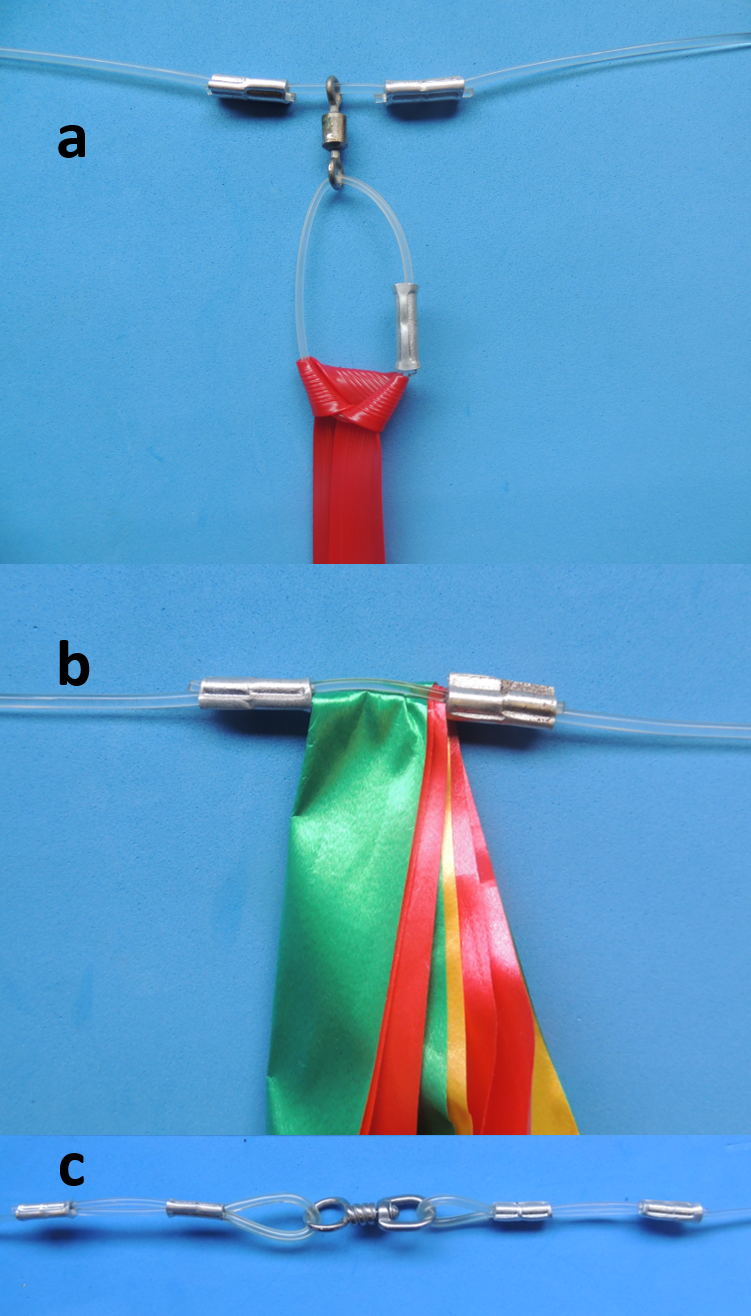

Supplement: S2 Fig — (TIF) [file pone.0184465.s005.tif]

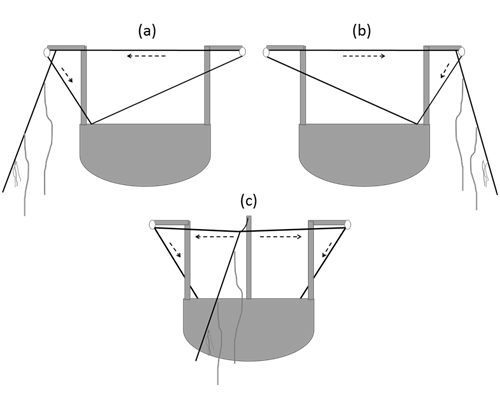

Supplement: S3 Fig — In the F/V the tori line was attached to a rope that was passed through the rings of the tori poles on each side of the vessel (S3a and S3b) and was shifted from the port side to the starboard and vice versa by pulling and fastening a lazy line. In the R/V, the tori line was attached to a pole located on the midline of the vessel and was shifted between the tori poles on each side by means of two lazy lines attached to the tori line via snaps (c). (TIF) [file pone.0184465.s006.tif]
